# Liquid Biopsy as a Diagnostic and Monitoring Tool in Glioblastoma

**DOI:** 10.3390/medicina61040716

**Published:** 2025-04-13

**Authors:** Ligia Gabriela Tataranu

**Affiliations:** 1 Department of Neurosurgery, Carol Davila University of Medicine and Pharmacy, 020021 Bucharest, Romania; ligia.tataranu@umfcd.ro; 2 Department of Neurosurgery, Bagdasar-Arseni Emergency Clinical Hospital, 041915 Bucharest, Romania

**Keywords:** glioblastoma, liquid biopsy, biomarkers, CTC, cfDNA, cfRNA, extracellular vesicles, TEPs

## Abstract

Glioblastoma (GBM) is the most prevalent and aggressive primary central nervous system (CNS) tumor in adults. GBMs exhibit genetic and epigenetic heterogeneity, posing difficulties in surveillance and being associated with high rates of recurrence and mortality. Nevertheless, due to the high infiltrating ability of glioblastoma cells, and regardless of the considerable progress made in radiotherapeutic, chemotherapeutic, and surgical protocols, the treatment of GBM is still inefficient. Conventional diagnostic approaches, such as neuroimaging techniques and tissue biopsies, which are invasive maneuvers, present certain challenges and limitations in providing real-time information, and are incapable of differentiating pseudo-progression related to treatment from real tumor progression. Liquid biopsy, the analysis of biomarkers such as nucleic acids (DNA/RNA), circulating tumor cells (CTCs), extracellular vesicles (EVs), or tumor-educated platelets (TEPs) that are present in body fluids, provides a minimally invasive and dynamic method of diagnosis and continuous monitoring for GBM. It represents a new preferred approach that enables a superior manner to obtain data on possible tumor risk, prognosis, and recurrence assessment. This article is a literature review that aims to provide updated information about GBM biomarkers in body fluids and to analyze their clinical efficiency.

## 1. Introduction

Glioblastoma (GBM) is the most common malignant brain tumor, with most patients having a mean overall survival of 12 to 15 months [1,2,3,4]. For many years, glioblastoma was classified based on histological characteristics, including necrosis, the proliferation of microvascularization, and the capacity to infiltrate the surrounding tissues. GBMs were divided into primary, thought to appear de novo, and secondary, originating from a low-grade glioma [5]. Both types of GBMs manifest with mutually exclusive gene alterations, revealing different tumor entities with distinctive natural progression and prognosis [6]. According to the new 2021 classification, they are categorized as grade 4 gliomas in 90% of cases (called “glioblastoma IDH wildtype”), and some low-grade and anaplastic astrocytomas with similar mutational status (called “astrocytoma IDH-mutant”) in 10% of cases [7,8]. The new classification may help identify different oncogenic pathways of biologically distinctive tumor categories, helping clinical decision-making and developing new therapeutic strategies [9].

The current therapeutic strategy, according to the Stupp protocol, includes surgical maximal safe resection, radiotherapy, and chemotherapy with temozolomide. Despite this intensive therapy plan, there is a high risk of recurrence associated with high mortality. Deficient drug dispersion into the CNS and the development of chemotherapy resistance represent relentless challenges, resulting in a poor prognosis [10,11].

There is a constant need for additional information in the case of glioblastoma. After the initial treatment, the disease progression is supervised using serial magnetic resonance scans, which are non-invasive procedures with no known risks, but which unfortunately are not able to differentiate between pseudo-progression and recurrent disease consistently [12]. Additionally, MR images are not capable of sufficiently correlating the actual neoplastic disease burden, with micro-infiltrative processes being present outside the limits of radiological imaging [13].

So far, to achieve and confirm diagnosis, to classify tumoral subtypes, identify tumor-specific mutations, and to lead to personalized therapeutic protocols, tissue samples were obtained from tumor biopsies [14]. This type of procedure is usually invasive and challenging, sustaining an increased risk of bleeding, infection, and the possibility of producing significant brain lesions, which can cause critical neurological deficits [15]. Other disadvantages of these procedures are the limitations in acquiring high-quality tumor samples. To study tumor response and relapse, it is important to obtain tumor samples regularly [16]. Due to the intratumoral heterogeneity, acquiring tumor samples representative of the entire tumor profile is limited in invasive procedures [17], and in most cases, are not able to fully represent the intricacies of the disease [18]. The material obtained is often insufficient to realize all essential molecular tests needed for diagnosis [19]. In addition, in multifocal lesions, because tumors suffer a continuous evolution both spatially and temporally in response to treatment, several biopsies could be needed [16]. Due to the limitations presented by tissue biopsies, biological fluids are being studied lately to find a new method capable of helping in the diagnosis and follow-up of cancer patients.

This study aims to summarize the available data about recent progress on glioblastoma biomarkers in various body fluids that can be identified and analyzed using the emerging minimally invasive liquid biopsy technique. This technique could be a potential instrument for early and accurate diagnosis, prognostic assessment, initial treatment stratification, and treatment response evaluation.

## 2. Liquid Biopsy

**Figure 1:**
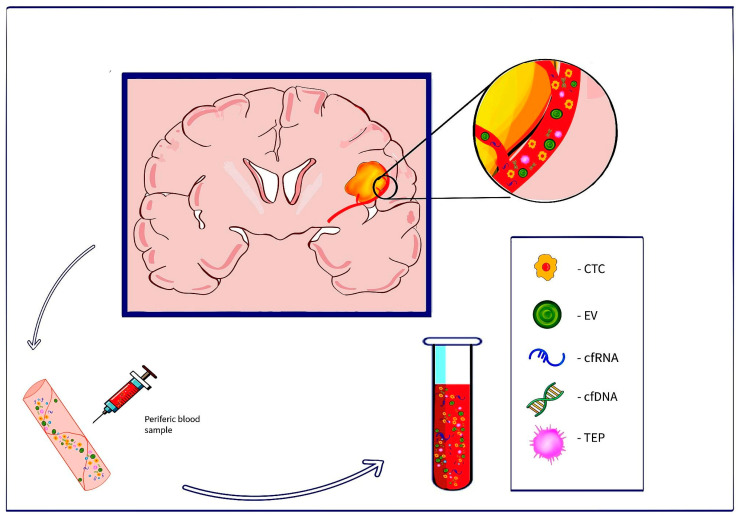
Examples of circulating biomarkers released into the bloodstream by the tumor mass. They can be collected and analyzed for tumor-specific changes.

Liquid biopsy was developed as a minimally invasive method capable of aiding in diagnosis, molecular profiling, and tumor monitoring, providing insights into risk, prognosis, and recurrence in oncological patients [17,20]. The term “Liquid biopsy” was first introduced in 2010 by Alix-Panabières and Pantel [21,22], describing circulating tumor cells. Liquid biopsy aims to detect and quantify tumor-derived content—cell-free nucleic acids DNA/RNA, extracellular vesicles (EVs) such as exosomes and microvesicles, that can encapsule DNA, RNA and other particles, circulating tumor cells (CTCs), proteins, metabolites, and tumor-educated platelets (TEPs), that can travel to distant organs and can be identified in body fluids—blood, cerebrospinal fluid (CSF), urine, saliva, fluid from pleural effusions, etc. (Figure 1) [23,24,25,26]. These tumor cell components can be used as cancer biomarkers, offering comparable and even more complete data than a tissue biopsy, identifying the site of origin, and helping in tumor progression and treatment efficiency monitoring [14]. Liquid biopsy has the potential to overcome the limitations of neuroimaging and tissue-based methods to identify early recurrence and differentiate tumor progression from pseudoprogression. Because of the ability to study specific tumor-related elements released in body fluids, liquid biopsy is used in several tumor types nowadays, offering important information regarding diagnostics, prognostics, and real-time updates on tumor status [27]. This game-changing technique has the potential to reform precision oncology and individualized treatment.

### 2.1. Cell-Free Nucleic Acids (cfNAs)

Numerous types of cells, including tumor cells, discharge DNA and RNA fragments into bodily fluids. These fragments are liberated through necrosis, apoptosis, and active secretion. These types of unbound nucleic molecules were first discovered and described in 1948 by Mandel and Metais [28]. cfNAs are derived from both normal and tumoral cells and are represented by double-strand cell-free DNA (cfDNA) and cell-free RNA (cfRNA), which include mRNA and non-coding RNA, like microRNA (miRNA), long non-coding RNA (lncRNA), and circular RNA (circRNA), that participate in gene regulation [29,30]. The identification of cfNAs in body fluids is possible using a few different techniques, such as next-generation sequencing (NGS), polymerase chain reaction (PCR), and droplet digital PCR (ddPCR). These methods allow the amplification and analysis of specific sequences of nucleic acids, facilitating the recognition of mutations, gene expression patterns, and genetic profiles that are tumor-specific [31,32,33]. Various investigation studies revealed the promising future of using cfNAs as diagnosis and prognosis biomarkers in glioblastoma [29].

In most cases, the concentration of cell-free nucleic acids in body fluids is low, making their detection and analysis very difficult. Special detection methods like ddPCR and NGS are needed to deal with this problem [24,31]. The existence of non-tumor cell-derived nucleic acids can complicate the data analysis. The amount and distribution of cfNAs can also be affected by the glioblastoma heterogeneity, influencing their diagnostic and prognostic utility [31]. New detection methods with higher accessibility and more cost-effectiveness must be found to further promote the use of cfNAs as biomarkers for GBM [29,34].

#### 2.1.1. Cell-Free DNA (cfDNA)

**Figure 2:**
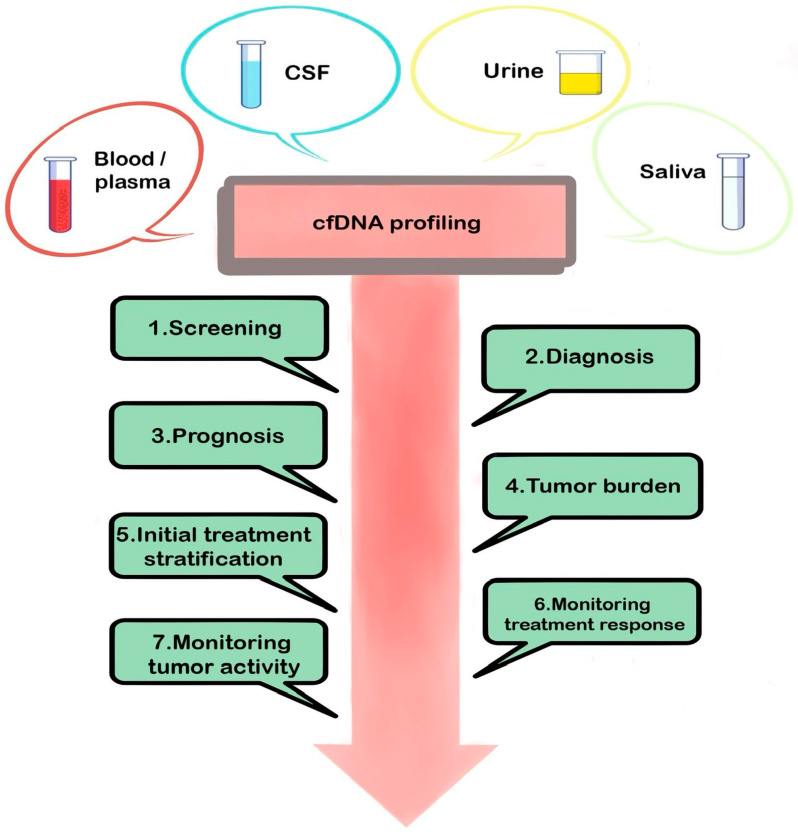
Profiling cancer-associated genetic alterations in cell-free DNA obtained from body fluids, such as blood or plasma, CSF, urine, and saliva, offers the possibility of screening healthy or at-risk asymptomatic patients for early detection, diagnosis, and treatment of cancer.

cfDNA is represented by short fragments (usually 130–180 base pairs) and double-stranded DNA, is not associated with cells or cellular fragments [35,36], and is usually present in the bloodstream, with physiological oscillations in response to inflammation, cellular injury, and in reply to exercise [37]. Circulating tumor DNA (ctDNA) is released into the bloodstream by tumor cells undergoing necrosis, apoptosis, and/or active cellular secretion [38]. In oncological patients, circulating tumor DNA (ctDNA) discarded by cancer cells represents only a part of the total cfDNA [39]. ctDNA was first described by Stroun et al. in 1989 [40]. Although the ctDNA proportion in the total cfDNA is low, it can still be detected and offer information about the tumor molecular profile [41,42]. Profiling of genetic alterations in cfDNA holds considerable promise as a biomarker with multiple functions in oncology, including diagnosis, prognosis, tumor burden, treatment stratification, monitoring response to treatment, and monitoring progression (Figure 2).

Using NGS can help detect mutations and determine the sensitivity and resistance to targeted therapies [43,44,45,46]. Since both ctDNA [47,48] and cfDNA [49,50] have a short half-life in the plasma, they have been considered real-time biomarkers of tumor burden, especially due to their potential as minimally invasive surveillance methods. Using a serum cfDNA epigenetic framework, Johnson et al. were able to make the distinction between IDH-mutant and IDH-wildtype GBM, showing that cfDNA’s unique epigenetic features can mark the difference between tumors with similar origins but with separate clinical trajectories [51]. The corticosteroids used in edema management could influence the levels of cfDNA identified in liquid biopsies [52]. Because of the steroids’ ability to lower vascular permeability, the amount of cfDNA liberated into the bloodstream may be lower, affecting the sensitivity of ctDNA detection and influencing the precision of real-time tumor surveillance during treatment [52].

The most proper biological sample for cfDNA evaluation in brain tumors is CSF, and CSF is obtained through an invasive procedure, which is very difficult to use as a routine analysis to oversee the response to treatment [53]. Another biological fluid that poses certain advantages is saliva. It is easy to collect, transport, and store because it does not coagulate. Nevertheless, the saliva composition (water, mucins, enzymes, proteins, food residues, or other particles) can influence the analysis results [54]. cfDNA displays a shorter half-life in urine samples because of elevated Deoxyribonuclease (DNase) I and II activity. New effective ways of storage and processing are still under investigation to lower the risks of cfDNA loss from urine samples [55,56].

Glioblastoma presents with a lower level of cfDNA than other solid tumors, making identifying circulating tumor DNA (ctDNA) particularly difficult. This can indicate that the blood–brain barrier may restrict the ctDNA from entering the systemic circulation [36]. Studies that investigated the correlation between the blood–brain-barrier (BBB) disruption, tumor-associated macrophages (TAMs), and the concentration of cfDNA and ctDNA in plasma from glioblastoma patients reported that there is a positive interconnection between the cfDNA plasma concentration and the volume of the tumor that displays elevated K_trans_ (the volume transfer constant that represents the efflux rate of substances from blood plasma and the surrounding extracellular space) and K_ep_ (the rate at which contrast substances leaves the extracellular space returning into the bloodstream). It was also reported that there is an association between the tumor vessel size, TAM density, and levels of plasma cfDNA. Higher CD68+ macrophages perivascular density was linked with lower BBB permeability and reduced detection rate of somatic mutations in plasma from glioblastoma patients [57].

In a study by Piccioni et al., 222 GBM patients were enrolled in a cohort, and a highly sensitive and specific NGS panel was applied. ctDNA mutations were reported in 55% of plasma samples [58]. ctDNA can fully portray the glioblastoma genome profile. The rate of detection of ctDNA fluctuates according to the tumoral histopathological stage [58,59]. The most common gene mutations that were found in the patient’s plasma using NGS were TP53 (tumor protein p53), NF-1, EGFR1(epidermal growth factor receptor 1), MET, APC, PDGFRA (platelet-derived growth factor receptor alpha), ERBB2, and EGFR (epidermal growth factor receptor) [58,60]. Methylation of the MGMT (O6-Methylguanine-DNA Methyltransferase) gene promoter was detected in serum ctDNA [61]. Patients with an increased methylation status responded better to treatment with alkylating agents.

A prospective study has been performed on 42 glioblastoma patients to detect the possible clinical applications of cfDNA. They were investigated, and the capacity of cfDNA was demonstrated to be used as a biomarker of prognostic and tumor burden and as a tool to differentiate between proper tumor progression and pseudoprogression. Plasma cfDNA was evaluated at baseline before initial surgery and longitudinally during chemoradiotherapy. Results indicated a higher plasma cfDNA concentration in newly diagnosed GBM patients compared to age-matched healthy individuals. It was also observed that higher plasma cfDNA concentrations were linked to a poorer prognosis. Targeted NGS panels were used to analyze somatic tumor mutations in plasma samples collected before surgical resection. The study was able to determine the utility of serial and longitudinal collection of plasma cfDNA in new cases of glioblastoma, offering quantitative evidence [35].

A study performed by Fontanilles et al. in a cohort of 49 newly diagnosed glioblastoma patients treated with concomitant temozolomide (TMZ) and radiotherapy (RT), followed by a TMZ maintenance phase, evaluated the ability of cfDNA and ctDNA to be used as biomarkers for disease surveillance. Plasma samples were collected at baseline, before RT-TMZ, after adjuvant TMZ, and/or at the time of progression in case of progressive disease. The cfDNA concentration was determined using a fluorometric method, and the ctDNA was detected using targeted ddPCR. Important fluctuations in cfDNA concentration were detected amid treatment, with lower levels after surgical treatment and higher levels throughout tumor regression. The study illustrated the ability of cfDNA to be used as a prognostic marker for disease progression in glioblastoma patients. More studies are needed to demonstrate the prognostic implications of cfDNA concentration fluctuations pre- and post-treatment [62].

Bagley et al. observed the relationship between plasma cfDNA concentration and survival outcome in a study of 62 patients presenting with IDH wild-type GBM. Poorer PFS (progression-free survival) and OS (overall survival) were associated with high pre-operative cfDNA plasma levels. Post-hoc evaluation of the modification in cfDNA values post-chemoradiotherapy compared to the levels before surgery was associated with inferior PFS and OS [63].

Because of the facts that CSF is both arduous to collect and associated with important discomfort for the patient, Mouliere et al. supervised a study that was meant to verify the feasibility of the use of cfDNA detection in plasma and urine as a non-invasive approach designed for longitudinal sampling for glioblastoma patients. Using tumor-guided sequencing, they analyzed the tumor mutational burden and detection rate of ctDNA in CSF, plasma, and urine. Afterwards, they developed personalized sequencing techniques that were applied to analyze mutations that were private to/or shared between multiple regions of the same tumors to have greater confidence in the mutation calls. The mutations were used to generate targeted panels for high-depth sequencing of CSF, plasma, and urine. The authors identified size differences between mutant and non-mutant DNA in plasma, urine, and CSF samples, and it was observed that cfDNA fragments originating from tumors were shorter than non-mutant cfDNA. The personalized sequencing techniques could revolutionize the detection and monitoring of glioblastoma patients, offering genetic information, facilitating personalized treatment approaches, and contributing to the real-time monitoring of the tumor dynamics. The non-invasive character of plasma and urine could allow for more regular and less restrictive monitoring for GBM patients, providing benefits in the follow-up period in combination with imaging [64].

When comparing the results from genomic profiling of tumor samples obtained from tissue biopsy and of DNA material obtained from other sources through liquid biopsy, i.e., ctDNA and CTCs, it was observed that various mutations were found only in the tissue material in some cases [31], while in other cases, ctDNA was able to detect mutations that were not present in the tumoral material collected during the tissue biopsies [65]. This phenomenon can be explained by the GBM heterogeneity and by the fact that there can be certain differences among liquid and tissue biopsy results based on the nature of the tumor, the quantitative and qualitative characteristics of the cfDNA and CTCs, and the techniques used for isolation and sequencing [66].

DNA and RNA sequencing are important tools that can aid in a better perception of tumor progression and in determining new mutations. The information obtained can be utilized to perfect new targeted therapies with higher mutation specificity. Sequencing can also be used to observe tumor fluctuations over time, like the appearance of new mutations or the development of drug resistance [67].

#### 2.1.2. Cell-Free RNA (cfRNA)

The GBM molecular heterogeneity hinders advances in investigating new and effective diagnostic methods and treatment approaches. Several RNA molecules could be used for cancer diagnosis, progression, and treatment response surveillance. GBM RNA markers can be found in circulating cell-free RNA, circulating exosomes, platelets, and CTCs that are identified either in peripheral blood or in CSF samples, saliva, or urine [23,68,69,70]. Cell-free RNAs are represented by RNA fragments that are classically differentiated into coding and noncoding RNAs (ncRNAs), although the binary classification of coding vs. noncoding is now outdated. microRNAs (miRNAs) are a group of small non-coding RNAs with a transcript length of about 22 nucleotides [71,72]. It has been demonstrated that miRNAs found in CSF represent a more significant diagnostic instrument, with higher sensitivity and specificity than their serum levels [73].

Circulating cell-free mRNA (ccfmRNA) was found in the plasma of cancer patients and studied as a noninvasive biomarker for glioma diagnosis and disease monitoring. mRNA expression can provide several molecular tumoral characteristics, like subtype, grade, and prognosis [74].

The cell-free miRNAs present important properties like high stability in human body fluids because of their binding to specific proteins or inclusion into extracellular vesicles, such as exosomes, microvesicles, and apoptotic bodies, and resistance to degradation in harmful conditions. These properties give them important biomarker abilities [75]. Studies have demonstrated that in GBM patients, miR-222 and miR-221 were overexpressed compared to healthy subjects. Raised miRNA levels were associated with inferior survival outcomes [76]. Another significant discovery was the downregulation of serum miR-100 expression levels in GBM patients compared with the control group, with higher serum miR-100 levels observed after treatment, suggesting that miR-100 values could be used as an indicator of treatment response [77]. Another study identified a correlation between GBM grade and three downregulated miRNAs—miR-128, miR-485-3p, and miR-342-3p. These were proposed as biomarkers for evaluating tumor grading and response to treatment [78].

lncRNAs (long noncoding RNAs) are RNA molecules with a length equal to or greater than 200 nucleotides. They could be used as promising diagnostic and prognostic biomarkers in glioblastoma [79]. It was determined that they are dynamic contributors to various biological pathways, like immune response and neural lineage commitment, and important modulators in disease processes [80]. The analysis of blood samples from glioblastoma patients exposed upregulation in the expression levels of various lncRNAs, such as HOTAIR (HOX Transcript Antisense RNA), MALAT1(metastasis-associated lung adenocarcinoma transcript-1), H19 (H19 imprinted maternally expressed transcript), ANRIL (antisense noncoding RNA in the INK4 Locus), LINC00641 (long intergenic non-protein coding RNA 641), LINC00565 (long intergenic non-protein coding RNA 565), SAMMSON (survival-associated mitochondrial melanoma specific oncogenic noncoding RNA), and downregulation in the expression of GAS5 (growth arrest specific 5) [81,82,83,84,85]. lncRNAs may play critical regulatory roles in diverse cellular processes, such as chromatin remodeling, transcription, posttranscriptional processing, and intracellular trafficking.

### 2.2. Extracellular Vesicles

Extracellular vesicles are represented by a heterogeneous group of cell-derived membrane-surrounded particles that carry bioactive molecules and deliver them to recipient cells. Their sizes range from 30 nm to 10 μm, including exosomes, microvesicles, and apoptotic bodies, and various cell types release them. The biomolecules within EVs are surrounded by a lipid bilayer, protecting them from enzyme degradation and allowing them to cross the blood–brain barrier [86]. They are easy to obtain from blood, urine, CSF, ascites, amniotic fluid, and seminal fluid, reflecting biological status and tumor condition, making them an encouraging research objective [87,88,89,90]. Studies have demonstrated that EVs are important as mediators of intercellular communication between glioblastomas and their microenvironment [91]. EV constituents, i.e., nucleic acids, proteins, lipids, and metabolites, can be relocated to adjacent or remote cells through a straight connection with the cellular membrane, fusion, or internalization [92,93]. In cancers, they are involved in various processes, including tumor–microenvironment interactions, cell proliferation, migration, immunosuppression, and intercellular communication [94,95,96,97,98]. Several investigations observed an interconnection between the number of circulating EVs and tumor burden, as well as OS and recurrence, implying that EVs can and should be used as biomarkers [99]. The EVs released by the glioblastoma and stromal cells favor intercellular communication within the tumor microenvironment.

EVs can be identified in plasma samples from patients using different methods, such as ultracentrifugation, density gradient ultracentrifugation, differential ultracentrifugation, size-based isolation techniques like ultrafiltration, and size exclusion chromatography. The technique is chosen based on the study’s needs and available resources. All of these techniques are different in terms of performance and purity [100]. Using mass spectrometry, the EVs’ cargo can be characterized. The results provide data about molecular properties and glioblastoma cell subtypes.

A study by Döring et al. included 36 glioblastoma patients with different disease statuses. It was designed to determine EV concentration at different stages of the disease, as in pre- and post-surgery, and during follow-up for patients with stable disease status and patients in progression. It was observed that higher EV concentrations were present in patients with progressive disease, suggesting that EV levels may be correlated with disease status, having the ability to be used as a potential biomarker for detecting early recurrence [101,102,103]. The study also aimed to correlate EV concentration with tumor volume, measured using magnetic resonance scans, but failed to demonstrate such a relationship. Although data on non-cerebral cancers, such as breast cancer, revealed the existence of a correlation between EV concentration and tumor size, in the case of glioblastoma, the fact that the immune-privileged environment of the brain is less permeable may be responsible for the lack of correlation [104]. The study attempted to find an association with systemic inflammatory markers, such as leukocyte count and C-reactive protein, in pursuance of results demonstrating the utility of EV concentration as a possible predictor and biomarker. The investigators observed an association between EV levels and leukocyte count, with higher GBM burden being most probably linked with a cancer-induced pro-inflammatory environment [105].

Rosas-Alonso et al. evaluated the clinical use of MGMT methylation in small extracellular vesicles (sEV)-based liquid biopsy as an instrument for glioblastoma patient management and the benefits of sEV as a predictive biomarker and disease surveillance instrument in patients with IDH-wildtype GBM. sEV include all EVs with a diameter smaller than 200 nm, including exosomes and small microvesicles. The study had consistent results with tissue-based findings, having an exceptional sensitivity of 85.7% for detecting MGMT methylation in liquid biopsy. The authors suggest that the assessment of sEV-DNA could greatly help monitor disease progression in IDH-wildtype patients, offer important insights into tumor heterogeneity, and detect GBM biomarkers [106].

#### 2.2.1. Exosomes

Exosomes are the smallest extracellular vesicles (30–100nm) discovered in 1983 by Pan and Johnstone [107]. The molecular mechanisms that are the basis of the processes of exosome biogenesis are not yet entirely understood. Exosomes are created through endocytosis at the plasma membrane. This process leads to the creation of multivesicular endosomes (MVEs), followed by the birth of exosomes [108,109].

Exosomes are essential components of the intercellular transfer mechanism of bioactive molecules. They are also involved in several pathophysiological processes, such as tumor invasion, angiogenesis, proliferation, signaling-pathways modulation, immune evasion, and treatment resistance, all contributing to tumor progression.

Exosomes can emerge from both cancerous and non-cancerous cells, reflecting the molecular properties of their origin cells. Because they can incorporate DNA, RNA, and several proteins capable of reflecting a cancerous state and influencing cellular processes, they are considered essential cancer biomarkers [110,111]. They can penetrate the BBB and other body areas, and reach different body fluids, where they are identified with the help of a liquid biopsy [112,113].

ncRNAs, which include miRNAs, are included in the exosomal load, supporting the pathophysiology of cancer and modulating glioma-related processes [114,115]. miRNAs are plentiful in exosomes, stable, and easy to access in body fluids, making them a good option as biomarkers in gliomas [108,116]. Some particular exosomal miRNAs, like miR-19b-3p, miR-183-5p, and miR-323a-3p, have been studied as possible biomarkers found in the serum of glioblastoma patients. It was discovered that they regulate specific genes and pathways partly responsible for the malignant properties of the glioblastoma cells. Targeted regulation of these exosomal miRNAs could be a promising, effective therapeutic approach for glioblastoma [117].

Exosomes have multiple benefits as liquid biopsy diagnostic biomarkers for cancers. However, there are still many obstacles to the clinical use of exosomes, including the lack of standardized protocols for exosome isolation. Studies are in progress, demanded by the constant need for better diagnostic and treatment options [111,118,119,120,121].

#### 2.2.2. Microvesicles

Microvesicles (MVs) are a distinct population of vesicles (0.1–1.0 µm) that arise through budding from the cellular membrane. It was noticed that cancer cells release a significant number of MVs in comparison with normal cells [121]. MVs incorporate parts of the original cell, like cellular membranes, mRNA, cytoplasmic constituents, and several proteins. This helps intercellular communication and determines cellular behavior and functions [90,122]. MVs are collected from plasma and analyzed using flow cytometry [123]. In a study performed on GBM patients by Simionescu et al., it was proposed that identifying biomarkers such as specific miRNAs and the MVs that carry them is important for understanding the progression, recurrence, and treatment response in glioblastoma. Circulating MVs were isolated from the patients’ plasma before and after surgery, as well as from the healthy control subjects’ plasma. Results have shown a reduction of the MV’s parameters (number, EGFRvIII, and EpCam—epithelial cell adhesion molecule) after surgical treatment. The expression of miR-106 b-5p, miR-486-3p, and miR-766-3p was upregulated, while miR-30d-5p levels were downregulated; after surgery, all of them were restored to control-like levels. The study’s results also suggested that miR-625-5p may be used as a regression or recurrence biomarker in glioblastoma [124].

### 2.3. CTCs—Circulating Tumor Cells

CTCs are malignant cells that originate and separate themselves from the primary tumor or metastatic deposits [125]. They were first identified in 1869 by Thomas Ashworth [126,127]. CTCs offer a less invasive method of acquiring tumor samples to detect cancers. These cells enter the bloodstream or the lymphatic system and can offer important information regarding the genetic and phenotypic aspects of the tumor. The assessment of the molecular profile of these cells may offer a better understanding for clinicians, providing support in the detection of metastasis or recurrence, supervising disease progression, and evaluating the efficiency of the treatment. CTCs are relatively rare compared to other blood cells, the stage and type of tumor being an influencer of their presence [29,68]. The existence of CTCs in the circulatory system plays a significant role in the distant spread of cancer, being associated with high mortality due to malignant tumors [128]. Essential attributes like the epithelial–mesenchymal transition (EMT) and dormancy guarantee their survival in the bloodstream. They also present resistance to radiation and chemotherapy, and the capacity to metastasize and evade the anti-cancer immune responses. CTCs that persist in the bloodstream are considered the most aggressive cancer cells. Nevertheless, it was observed that most of the CTCs are rapidly discarded from the bloodstream, implying a reduced capacity to metastasize [129]. They enter the bloodstream through intravasation and exit through extravasation into other bodily fluids. Their presence is revealed through various techniques such as immunocytochemistry (where specific cell surface markers are labeled), flow cytometry (where cells are sorted based on size and the different surface markers), and microfluidic devices (CTCs are isolated based on size and specific antibody affinity). These procedures aim to evaluate CTC’s molecular and phenotypic profiles [29].

CTCs in bodily fluids in glioblastoma patients are connected with disease progression, treatment response, and patient survival, showing great potential for using CTCs as diagnostic and prognostic biomarkers. Higher numbers are being linked to poorer prognosis and diminished overall survival. Variation of the CTCs number counts during treatment can reflect the overall treatment success and the probability of recurrence [31]. Glioblastoma-derived CTCs present stem cell characteristics that are responsible for new local tumor formation and recurrence [130,131].

A certain number of methods are used to identify CTCs in GBM. Gradient centrifugation can isolate CTCs from red blood cells alone or followed by targeted removal of white blood cells using CD45. To probe for aneuploidy, especially targeting the chromosome 8 centromere, either immunocytochemical staining using GBM-specific markers, like GFAP (glial fibrillary acidic protein), is executed afterward, or fluorescence in situ hybridization (FISH). Another technique requires using green fluorescent protein under the control of the telomerase (hTERT) promoter through viral transduction to recognize cancer cells for CTC detection [132].

Investigating CTCs can offer important genomic data that mirrors the tumoral landscape. Telomerase analysis was utilized to detect CTCs in glioblastoma patients. It was demonstrated that patients with a good response to treatment had a lower count of CTCs after therapy. A study performed by MacArthur et al. uncovered that CTCs were present in 72% of patients before radiotherapy, compared to 8% after treatment [133]. Some studies that used GFAP (glial fibrillary acidic protein) to detect GBM CTCs in isolated peripheral mononuclear cells demonstrated that only 20% of the patients had CTCs in the peripheral blood samples [25,133,134,135,136]. The anti-glioma aptamers, which are nucleic acid molecules that have high affinity and selectivity, and bind solely to their objectives, Gli-233 and Gli-55, have been analyzed in liquid biopsies to identify CTCs, which can enhance diagnostic specificity [137,138]. Glioblastoma-derived CTCs present with EGFR amplification, which is linked to aggressiveness and the presence of EGFRvIII, and higher levels of SERPINE1 (serpin family e member 1), VIM (vimentin), TGFB1(transforming growth factor beta 1), and TGFBR2 (transforming growth factor beta receptor 2) genes, which are linked to the mesenchymal subtype [135,139].

The techniques used nowadays to detect GBM CTCs lack specificity and demand considerable microscopic screening to discover GBM markers among millions of cells. The possibility of developing antibody cross-reactivity or physical co-segregation of CTCs with blood cells causes the loss of CTCs, interfering with the use of negative enrichment techniques. The use of standardized CTC isolation techniques is essential to guarantee the reproducibility and comparability of outcomes across different investigation studies and clinical settings [132].

### 2.4. TEPs

Tumor-educated platelets (TEP) suffer a tumor-induced biomolecule transfer process called “education”. They receive tumor-associated molecules, resulting in platelets with different behavior [140]. Mutant RNA is transferred into blood platelets by cancer cells. During tumor progression, the platelets express altered spliced RNA profiles. These alterations can be caused by different mechanisms, such as the tumor-induced alteration of RNA that is transferred by megakaryocytes into platelets; platelet RNA alternative splicing tumor, stromal, or immune cells-derived events; altered platelet turnover; and the evolution of differential subpopulations of platelets [141]. It was observed that the genetic material included within platelets is somewhat more durable [142]. A microarray analysis detected that TEPs exhibit arrested tumor-derived EVs that present with EGFRvIII mutant RNA. EGFRvIII mutation was identified in 80% of GBM cases, but was absent in healthy control subjects [143].

Due to their high abundance, easy isolation, and the high-quality RNA they contain, TEPs are more advantageous to use over other blood-based approaches. Using RNA derived from TEPs could add value to the process of early diagnosis of cancer, and the process of non-invasive monitoring [144]. In a study conducted by Sol et al., the authors found that the TEP RNA signature could differentiate GBM patients from healthy asymptomatic controls with a detection accuracy of 95%. The study also demonstrated that TEPs could differentiate false-positive progression from actual progression with an accuracy of 85%. The study concluded that TEPs are a potentially minimally invasive biosource for blood-based diagnosis and disease monitoring in glioblastoma [145].

## 3. Future Directions

Currently, there is no clinical application for circulating biomarkers in managing glioblastoma patients. Using each type of biomarker, such as ctDNA, mRNAs, CTCs, EVs, and others, demonstrates distinct advantages and limitations. Notwithstanding the challenges presented by the detection and profiling of these biomarkers, they have the potential to supplement the available methodologies in the GBM management.

There are no clinically approved circulating biomarkers for GBM yet. The identification and use of each type of biomarker have specific limitations. One limitation is shared among most biomarkers and is represented by their short half-life in the blood, despite the fact that some of them are being protected from deterioration inside EVs [146,147].

Glioma ctDNA levels are minimal, probably because of the restriction posed by the BBB. The short life of the ctDNA of less than 2.5 h is another limitation for using this biomarker. The fact that ctDNA is released mainly by apoptotic and necrotic cells makes this biomarker a representative of only a subpopulation of tumor cells. The levels of ctDNA in biological fluids may also be influenced by physiological and non-tumor conditions like inflammation and autoimmune diseases, stroke, or traumatic brain injury, making it harder to distinguish tumor-specific ctDNA. Among the available detection methods, ddPCR and NGS are the most promising, but there is still a need for more studies on their use as routine procedures. The costs of using NGS as a routine screening method are very high, yet another limitation for the ctDNA detection [147].

cfRNA is released in small amounts and is very susceptible to degradation by RNases in the bloodstream. The cfRNA levels are determined by inflammatory and systemic conditions and environmental factors like diet and smoking. Differentiating between GBM-derived RNA and non-tumoral RNA is very difficult. The most promising detection methods for clinical use are represented by RT-qPCR (Reverse Transcription Quantitative Polymerase Chain Reaction) and ddPCR. NGS is very useful for broad miRNA profiling, but the high costs and the need for advanced bioinformatics make this detection method not feasible for clinical implementation [147].

Assessing extracellular vesicles, including exosomes, in biological fluids, especially CSF, is very difficult. Unlike blood, the CSF acquisition is more invasive, and the amount available is limited, restricting the total amount of EVs available for isolation and analysis. Because of the proximity to the glioblastoma, tumor-derived EVs are probably more abundant in CSF than blood and can reflect molecular changes earlier. EVs and exosomes have a short half-life of less than 30 min, limiting the time window for detection. Time-sensitive processing is very important for detecting them. The high amount of EVs released by non-tumoral cells makes distinguishing the tumoral ones even harder. There are no standardized methods for isolating them yet. Mass spectrometry and RNA sequencing are highly sensitive, but the high costs and technical challenges make them not feasible for routine usage. CSF-derived EVs could be used as glioblastoma biomarkers for glioblastoma detection, but improvements in isolation, stabilization, and detection technologies must be achieved [147].

CTCs isolation and quantification are impaired by the technical limitations. They are very rare in GBM, mostly because of the BBB, which limits their identification. They rapidly degrade in circulation because of the short half-life (1 to 2.4 h). Detection rates differ according to the techniques utilized for isolation. Due to the GBM intra- and intertumoral heterogeneity, it is difficult to determine a universal marker for CTCs. The currently available detection methods for CTCs (already in use for other types of cancers like the CellSearch System) have a low sensitivity for GBM. Other techniques like microfluidic-based capture still require further studies in order to be used for glioblastoma, while scRNA-Seq (single-cell RNA sequencing) is highly sensitive but very expensive [146,147].

Improvement of the available technologies for biomarker profiling and characterization is needed for their implementation in clinical practice. Using computational tools, such as artificial intelligence and machine learning, could help integrate all the data sets that emerge from future research. More large-scale clinical studies that assess the impact of these biomarkers on clinical outcomes are necessary to validate them for clinical application. These approaches could contribute to the identification of new biomarkers that might predict the treatment response in GBM, might help to identify the sensitivity and resistance to treatment mechanisms, and to detect promising therapy targets to guide treatment options [146,147].

## 4. Conclusions

Liquid biopsy could be a promising instrument in precision medicine for detecting, diagnosing, and monitoring disease progression and treatment response in glioblastoma patients. The capacity to analyze tumor-derived components from blood or other easy-to-obtain body fluids is very appealing in glioblastoma, especially because of the risks associated with surgery. The genetic and phenotypic components of GBM could be examined with the help of data provided by CTCs, cell-free nucleic acids, and EVs. cfDNAs can offer information about genetic and epigenetic alterations in GBM, while CTCs reveal tumor progression and treatment response data. Integrating all the data from the study of the biomarkers available in the body fluids can have diagnostic and prognostic utility. It might be useful in developing advanced therapeutic plans and managing the disease.

Because of the high costs of the detection techniques and low concentration in early-stage cancers, the use of cfDNA as a screening tool is still under debate. It could be used with great results to track tumor evolution, treatment resistance, recurrence, and to assess the prognosis by analyzing the MGMT promoter methylation status. cfRNA are not yet reliable for early screening because of the lack of tumoral standardized cfRNA markers and the low levels available in biological fluids. EVs can potentially be used as a screening biomarker if new isolation and detection methods are developed. CTCs are not convenient for early cancer detection because of their rarity and technical limitations.

Using the information acquired with liquid biopsies in clinical practice still faces several challenges. Because of the low concentrations of tumor-derived biomarkers, the variability of biomarker levels due to physiological and pathological factors, and the deficiency in standardized detection methods, their use for diagnosis is still controversial. The research must focus on developing and perfecting noninvasive detection techniques with high sensitivity and specificity, along with standardized protocols for isolation and characterization. More research is still necessary to validate the clinical value of these biomarkers in GBM.

## References

[1] King J.L., Benhabbour S.R. (2021). Glioblastoma Multiforme—A Look at the Past and a Glance at the Future. Pharmaceutics.

[2] Zhang C., Zhou W., Tan Y., Tian D., Zhong C. (2022). 5-Hydroxymethylcytosines in circulating cell-free DNA reveal a diagnostic biomarker for glioma. Heliyon.

[3] Ohgaki H., Kleihues P. (2007). Genetic Pathways to Primary and Secondary Glioblastoma. Am. J. Pathol..

[4] Ostrom Q.T., Cioffi G., Waite K., Kruchko C., Barnholtz-Sloan J.S. (2021). CBTRUS Statistical Report: Primary Brain and Other Central Nervous System Tumors Diagnosed in the United States in 2014–2018. Neuro-Oncology.

[5] Aldape K., Zadeh G., Mansouri S., Reifenberger G., von Deimling A. (2015). Glioblastoma: Pathology, molecular mechanisms and markers. Acta Neuropathol..

[6] Watanabe K., Tachibana O., Sato K., Yonekawa Y., Kleihues P., Ohgaki H. (1996). Overexpression of the EGF Receptor and *p53* Mutations are Mutually Exclusive in the Evolution of Primary and Secondary Glioblastomas. Brain Pathol..

[7] Louis D.N., Perry A., Wesseling P., Brat D.J., Cree I.A., Figarella-Branger D., Hawkins C., Ng H.K., Pfister S.M., Reifenberger G. (2021). The 2021 WHO Classification of Tumors of the Central Nervous System: A summary. Neuro-Oncology.

[8] Reuss D.E., Mamatjan Y., Schrimpf D., Capper D., Hovestadt V., Kratz A., Sahm F., Koelsche C., Korshunov A., Olar A. (2015). IDH mutant diffuse and anaplastic astrocytomas have similar age at presentation and little difference in survival: A grading problem for WHO. Acta Neuropathol..

[9] Aslan K., Turco V., Blobner J., Sonner J.K., Liuzzi A.R., Nunez N.G., de Feo D., Kickingereder P., Fischer M., Green E. (2020). Heterogeneity of response to immune checkpoint blockade in hypermutated experimental gliomas. Nat. Commun..

[10] Ammirati M., Chotai S., Newton H., Lamki T., Wei L., Grecula J. (2014). Hypofractionated intensity modulated radiotherapy with temozolomide in newly diagnosed glioblastoma multiforme. J. Clin. Neurosci..

[11] Mannas J.P., Lightner D.D., DeFrates S.R., Pittman T., Villano J.L. (2014). Long-term treatment with temozolomide in malignant glioma. J. Clin. Neurosci..

[12] Li Y., Ma Y., Wu Z., Xie R., Zeng F., Cai H., Lui S., Song B., Chen L., Wu M. (2021). Advanced Imaging Techniques for Differentiating Pseudoprogression and Tumor Recurrence After Immunotherapy for Glioblastoma. Front. Immunol..

[13] Garcia C.M., Toms S.A. (2020). The Role of Circulating MicroRNA in Glioblastoma Liquid Biopsy. World Neurosurg..

[14] Cohen J.D., Li L., Wang Y., Thoburn C., Afsari B., Danilova L., Douville C., Javed A.A., Wong F., Mattox A. (2018). Detection and localization of surgically resectable cancers with a multi-analyte blood test. Science.

[15] Chen M., Zhao H. (2019). Next-generation sequencing in liquid biopsy: Cancer screening and early detection. Hum. Genom..

[16] Perakis S., Speicher M.R. (2017). Emerging concepts in liquid biopsies. BMC Med..

[17] Skouras P., Markouli M., Kalamatianos T., Stranjalis G., Korkolopoulou P., Piperi C. (2023). Advances on Liquid Biopsy Analysis for Glioma Diagnosis. Biomedicines.

[18] Kurma K., Eslami-S Z., Alix-Panabières C., Cayrefourcq L. (2024). Liquid biopsy: Paving a new avenue for cancer research. Cell Adhes. Migr..

[19] Hirahata T., ul Quraish R., Quraish A.U., ul Quraish S., Naz M., Razzaq M.A. (2022). Liquid Biopsy: A Distinctive Approach to the Diagnosis and Prognosis of Cancer. Cancer Inform..

[20] Corcoran R.B., Chabner B.A. (2018). Application of Cell-free DNA Analysis to Cancer Treatment. N. Engl. J. Med..

[21] Pantel K., Alix-Panabières C. (2010). Circulating tumour cells in cancer patients: Challenges and perspectives. Trends Mol. Med..

[22] Pantel K., Alix-Panabières C. (2019). Liquid biopsy and minimal residual disease—latest advances and implications for cure. Nat. Rev. Clin. Oncol..

[23] Bauman M.M.J., Bouchal S.M., Monie D.D., Aibaidula A., Singh R., Parney I.F. (2022). Strategies, considerations, and recent advancements in the development of liquid biopsy for glioblastoma: A step towards individualized medicine in glioblastoma. Neurosurg. Focus.

[24] Jelski W., Mroczko B. (2021). Molecular and Circulating Biomarkers of Brain Tumors. Int. J. Mol. Sci..

[25] Westphal M., Lamszus K. (2015). Circulating biomarkers for gliomas. Nat. Rev. Neurol..

[26] Lone S.N., Nisar S., Masoodi T., Singh M., Rizwan A., Hashem S., El-Rifai W., Bedognetti D., Batra S.K., Haris M. (2022). Liquid biopsy: A step closer to transform diagnosis, prognosis and future of cancer treatments. Mol. Cancer.

[27] Jones J., Nguyen H., Drummond K., Morokoff A. (2021). Circulating Biomarkers for Glioma: A Review. Neurosurgery.

[28] Mandel P., Metais P. (1948). Nuclear Acids in Human Blood Plasma. C R Seances. Soc. Biol. Fil..

[29] Saenz-Antoñanzas A., Auzmendi-Iriarte J., Carrasco-Garcia E., Moreno-Cugnon L., Ruiz I., Villanua J., Egaña L., Otaegui D., Samprón N., Matheu A. (2019). Liquid biopsy in glioblastoma: Opportunities, applications and challenges. Cancers.

[30] Fontanilles M., Sanson M., Touat M. (2021). Liquid biopsy in neuro-oncology: Are we finally there?. Ann. Oncol..

[31] Müller Bark J., Kulasinghe A., Chua B., Day B.W., Punyadeera C. (2020). Circulating biomarkers in patients with glioblastoma. Br. J. Cancer.

[32] Palande V., Siegal T., Detroja R., Gorohovski A., Glass R., Flueh C., Kanner A.A., Laviv Y., Har-Nof S., Levy-Barda A. (2022). Detection of gene mutations and gene–gene fusions in circulating cell-free DNA of glioblastoma patients: An avenue for clinically relevant diagnostic analysis. Mol. Oncol..

[33] Li D., Bonner E.R., Wierzbicki K., Panditharatna E., Huang T., Lulla R., Mueller S., Koschmann C., Nazarian J., Saratsis A.M. (2021). Standardization of the liquid biopsy for pediatric diffuse midline glioma using ddPCR. Sci Rep..

[34] Sasmita A.O., Wong Y.P., Ling A.P.K. (2018). Biomarkers and therapeutic advances in glioblastoma multiforme. Asia. Pac. J. Clin. Oncol..

[35] Bagley S.J., Till J., Abdalla A., Sangha H.K., Yee S.S., Freedman J., Black T.A., Hussain J., Binder Z.A., Brem S. (2020). Clinical Utility of Plasma Cell-Free DNA in Adult Patients with Newly Diagnosed Glioblastoma: A Pilot Prospective Study. Clin. Cancer Res..

[36] Bettegowda C., Sausen M., Leary R.J., Kinde I., Wang Y., Agrawal N., Bartlett B.R., Wang H., Luber B., Alani R.M. (2014). Detection of Circulating Tumor DNA in Early- and Late-Stage Human Malignancies. Sci. Transl. Med..

[37] Leon S.A., Shapiro B., Sklaroff D.M., Yaros M.J. (1977). Free DNA in the serum of cancer patients and the effect of therapy. Cancer Res..

[38] Aarthy R., Mani S., Velusami S., Sundarsingh S., Rajkumar T. (2015). Role of Circulating Cell-Free DNA in Cancers. Mol. Diagn. Ther..

[39] Diehl F., Schmidt K., Choti M.A., Romans K., Goodman S., Li M., Thornton K., Agrawal N., Sokoll L., Szabo S.A. (2008). Circulating mutant DNA to assess tumor dynamics. Nat. Med..

[40] Stroun M., Anker P., Maurice P., Lyautey J., Lederrey C., Beljanski M. (1989). Neoplastic Characteristics of the DNA Found in the Plasma of Cancer Patients. Oncology.

[41] Thompson J.C., Yee S.S., Troxel A.B., Savitch S.L., Fan R., Balli D., Lieberman D.B., Morrissette J.D., Evans T.L., Bauml J. (2016). Detection of Therapeutically Targetable Driver Resistance Mutations in Lung Cancer Patients by Next-Generation Sequencing of Cell-Free Circulating Tumor DNA. Clin. Cancer Res..

[42] Newman A.M., Bratman S.V., To J., Wynne J.F., Eclov N.C.W., Modlin L.A., Liu C.L., Neal J.W., Wakelee H.A., Merritt R.E. (2014). An ultrasensitive method for quantitating circulating tumor DNA with broad patient coverage. Nat. Med..

[43] Diaz L.A., Williams R.T., Wu J., Kinde I., Hecht J.R., Berlin J., Allen B., Bozic I., Reiter J.G., Nowak M.A. (2012). The molecular evolution of acquired resistance to targeted EGFR blockade in colorectal cancers. Nature.

[44] Aggarwal C., Thompson J.C., Black T.A., Katz S.I., Fan R., Yee S.S., Chien A.L., Evans T.L., Bauml J.M., Alley E.W. (2019). Clinical Implications of Plasma-Based Genotyping with the Delivery of Personalized Therapy in Metastatic Non–Small Cell Lung Cancer. JAMA Oncol..

[45] Sacher A.G., Paweletz C., Dahlberg S.E., Alden R.S., O’Connell A., Feeney N., Mach S.L., Jänne P.A., Oxnard G.R. (2016). Prospective Validation of Rapid Plasma Genotyping for the Detection of *EGFR* and *KRAS* Mutations in Advanced Lung Cancer. JAMA Oncol..

[46] Lin K.K., Harrell M.I., Oza A.M., Oaknin A., Ray-Coquard I., Tinker A.V., Helman E., Radke M.R., Say C., Vo L.-T. (2019). *BRCA* Reversion Mutations in Circulating Tumor DNA Predict Primary and Acquired Resistance to the PARP Inhibitor Rucaparib in High-Grade Ovarian Carcinoma. Cancer Discov..

[47] Choudhury A.D., Werner L., Francini E., Wei X.X., Ha G., Freeman S.S., Rhoades J., Reed S.C., Gydush G., Rotem D. (2018). Tumor fraction in cell-free DNA as a biomarker in prostate cancer. JCI Insight.

[48] Wei T., Zhang Q., Li X., Su W., Li G., Ma T., Gao S., Lou J., Que R., Zheng L. (2019). Monitoring Tumor Burden in Response to FOLFIRINOX Chemotherapy Via Profiling Circulating Cell-Free DNA in Pancreatic Cancer. Mol. Cancer Ther..

[49] Valpione S., Gremel G., Mundra P., Middlehurst P., Galvani E., Girotti M.R., Lee R.J., Garner G., Dhomen N., Lorigan P.C. (2018). Plasma total cell-free DNA (cfDNA) is a surrogate biomarker for tumour burden and a prognostic biomarker for survival in metastatic melanoma patients. Eur. J. Cancer.

[50] Gangadhar T.C., Savitch S.L., Yee S.S., Xu W., Huang A.C., Harmon S., Lieberman D.B., Soucier D., Fan R., Black T.A. (2018). Feasibility of monitoring advanced melanoma patients using cell-free DNA from plasma. Pigment. Cell Melanoma Res..

[51] Johnson K.C., Verhaak R.G.W. (2021). Serum cell-free DNA epigenetic biomarkers aid glioma diagnostics and monitoring. Neuro-oncology.

[52] Aran V., de Melo Junior J.O., Pilotto Heming C., Zeitune D.J., Moura Neto V., Niemeyer Filho P. (2024). Unveiling the impact of corticosteroid therapy on liquid biopsy-detected cell-free DNA levels in meningioma and glioblastoma patients. J. Liq. Biopsy..

[53] Wang Y., Springer S., Zhang M., McMahon K.W., Kinde I., Dobbyn L., Ptak J., Brem H., Chaichana K., Gallia G.L. (2015). Detection of tumor-derived DNA in cerebrospinal fluid of patients with primary tumors of the brain and spinal cord. Proc. Natl. Acad. Sci. USA.

[54] Hyun K.-A., Gwak H., Lee J., Kwak B., Jung H.-I. (2018). Salivary Exosome and Cell-Free DNA for Cancer Detection. Micromachines.

[55] Yao W., Mei C., Nan X., Hui L. (2016). Evaluation and comparison of in vitro degradation kinetics of DNA in serum, urine and saliva: A qualitative study. Gene.

[56] Lee E.Y., Lee E.-J., Yoon H., Lee D.H., Kim K.H. (2020). Comparison of Four Commercial Kits for Isolation of Urinary Cell-Free DNA and Sample Storage Conditions. Diagnostics.

[57] Nabavizadeh S.A., Ware J.B., Guiry S., Nasrallah M.P., Mays J.J., Till J.E., Hussain J., Abdalla A., Yee S.S., Binder Z.A. (2020). Imaging and histopathologic correlates of plasma cell-free DNA concentration and circulating tumor DNA in adult patients with newly diagnosed glioblastoma. Neuro-Oncol. Adv..

[58] Piccioni D.E., Achrol A.S., Kiedrowski L.A., Banks K.C., Boucher N., Barkhoudarian G., Kelly D.F., Juarez T., Lanman R.B., Raymond V.M. (2019). Analysis of cell-free circulating tumor DNA in 419 patients with glioblastoma and other primary brain tumors. CNS Oncol..

[59] Zill O.A., Banks K.C., Fairclough S.R., Mortimer S.A., Vowles J.V., Mokhtari R., Gandara D.R., Mack P.C., Odegaard J.I., Nagy R.J. (2018). The Landscape of Actionable Genomic Alterations in Cell-Free Circulating Tumor DNA from 21,807 Advanced Cancer Patients. Clin. Cancer Res..

[60] Schwaederle M., Husain H., Fanta P.T., Piccioni D.E., Kesari S., Schwab R.B., Banks K.C., Lanman R.B., Talasaz A., Parker B.A. (2016). Detection rate of actionable mutations in diverse cancers using a biopsy-free (blood) circulating tumor cell DNA assay. Oncotarget.

[61] Tomei S., Volontè A., Ravindran S., Mazzoleni S., Wang E., Galli R., Maccalli C. (2021). MicroRNA Expression Profile Distinguishes Glioblastoma Stem Cells from Differentiated Tumor Cells. J. Pers. Med..

[62] Fontanilles M., Marguet F., Beaussire L., Magne N., Pépin L.-F., Alexandru C., Tennevet I., Hanzen C., Langlois O., Jardin F. (2020). Cell-free DNA and circulating TERT promoter mutation for disease monitoring in newly-diagnosed glioblastoma. Acta Neuropathol. Commun..

[63] Bagley S.J., Till J., Abdalla A., Sangha H.K., Yee S.S., Freedman J., Black T.A., Hussain J., Binder Z.A., Brem S. (2021). Association of plasma cell-free DNA with survival in patients with IDH wild-type glioblastoma. Neuro-Oncol. Adv..

[64] Mouliere F., Smith C.G., Heider K., Su J., van der Pol Y., Thompson M., Morris J., Wan J.C.M., Chandrananda D., Hadfield J. (2021). Fragmentation patterns and personalized sequencing of cell-free DNA in urine and plasma of glioma patients. EMBO Mol. Med..

[65] Fontanilles M., Marguet F., Bohers É., Viailly P.-J., Dubois S., Bertrand P., Camus V., Mareschal S., Ruminy P., Maingonnat C. (2017). Non-invasive detection of somatic mutations using next-generation sequencing in primary central nervous system lymphoma. Oncotarget.

[66] Liebs S., Eder T., Klauschen F., Schütte M., Yaspo M.-L., Keilholz U., Tinhofer I., Kidess-Sigal E., Braunholz D. (2021). Applicability of liquid biopsies to represent the mutational profile of tumor tissue from different cancer entities. Oncogene.

[67] Karczewski K.J., Snyder M.P. (2018). Integrative omics for health and disease. Nat. Rev. Genet..

[68] Tamai S., Ichinose T., Nakada M. (2023). Liquid biomarkers in glioma. Brain Tumor Pathol..

[69] Bustos M.A., Rahimzadeh N., Ryu S., Gross R., Tran L.T., Renteria-Lopez V.M., Ramos R.I., Eisenberg A., Hothi P., Kesari S. (2022). Cell-free plasma microRNAs that identify patients with glioblastoma. Lab. Investig..

[70] Kopkova A., Sana J., Machackova T., Vecera M., Radova L., Trachtova K., Vybihal V., Smrcka M., Kazda T., Slaby O. (2019). Cerebrospinal Fluid MicroRNA Signatures as Diagnostic Biomarkers in Brain Tumors. Cancers.

[71] Derrien T., Johnson R., Bussotti G., Tanzer A., Djebali S., Tilgner H., Guernec G., Martin D., Merkel A., Knowles D.G. (2012). The GENCODE v7 catalog of human long noncoding RNAs: Analysis of their gene structure, evolution, and expression. Genome Res..

[72] Senhaji N., Squalli Houssaini A., Lamrabet S., Louati S., Bennis S. (2022). Molecular and Circulating Biomarkers in Patients with Glioblastoma. Int. J. Mol. Sci..

[73] Gao F., Cui Y., Jiang H., Sui D., Wang Y., Jiang Z., Zhao J., Lin S. (2016). Circulating tumor cell is a common property of brain glioma and promotes the monitoring system. Oncotarget.

[74] Ita M.I., Wang J.H., Toulouse A., Lim C., Fanning N., O’Sullivan M., Nolan Y., Kaar G.F., Redmond H.P. (2022). The utility of plasma circulating cell-free messenger RNA as a biomarker of glioma: A pilot study. Acta Neurochir..

[75] Ortiz-Quintero B. (2016). Cell-free microRNAs in blood and other body fluids, as cancer biomarkers. Cell Prolif..

[76] Swellam M., Ezz El Arab L., Al-Posttany A.S., Said S.B. (2019). Clinical impact of circulating oncogenic MiRNA-221 and MiRNA-222 in glioblastoma multiform. J. Neuro-Oncol..

[77] Makowska M., Smolarz B., Romanowicz H. (2023). microRNAs (miRNAs) in Glioblastoma Multiforme (GBM)—Recent Literature Review. Int. J. Mol. Sci..

[78] Wang Q., Li P., Li A., Jiang W., Wang H., Wang J., Xie K. (2012). Plasma specific miRNAs as predictive biomarkers for diagnosis and prognosis of glioma. J. Exp. Clin. Cancer Res..

[79] Rynkeviciene R., Simiene J., Strainiene E., Stankevicius V., Usinskiene J., Miseikyte Kaubriene E., Meskinyte I., Cicenas J., Suziedelis K. (2018). Non-Coding RNAs in Glioma. Cancers..

[80] Zan X.-Y., Li L. (2019). Construction of lncRNA-mediated ceRNA network to reveal clinically relevant lncRNA biomarkers in glioblastomas. Oncol. Lett..

[81] Shen J., Hodges T.R., Song R., Gong Y., Calin G.A., Heimberger A.B., Zhao H. (2018). Serum HOTAIR and GAS5 levels as predictors of survival in patients with glioblastoma. Mol. Carcinog..

[82] Ahir B.K., Ozer H., Engelhard H.H., Lakka S.S. (2017). MicroRNAs in glioblastoma pathogenesis and therapy: A comprehensive review. Crit. Rev. Oncol. Hematol..

[83] Zhang J.-X., Han L., Bao Z.-S., Wang Y.-Y., Chen L.-Y., Yan W., Yu S.-Z., Pu P.-Y., Liu N., You Y.-P. (2013). HOTAIR, a cell cycle–associated long noncoding RNA and a strong predictor of survival, is preferentially expressed in classical and mesenchymal glioma. Neuro-Oncology.

[84] Tan S.K., Pastori C., Penas C., Komotar R.J., Ivan M.E., Wahlestedt C., Ayad N.G. (2018). Serum long noncoding RNA HOTAIR as a novel diagnostic and prognostic biomarker in glioblastoma multiforme. Mol. Cancer.

[85] Pokorná M., Černá M., Boussios S., Ovsepian S.V., O’Leary V.B. (2024). lncRNA Biomarkers of Glioblastoma Multiforme. Biomedicines.

[86] Cordonnier M., Chanteloup G., Isambert N., Seigneuric R., Fumoleau P., Garrido C., Gobbo J. (2017). Exosomes in cancer theranostic: Diamonds in the rough. Cell Adh. Migr..

[87] Cilibrasi C., Simon T., Vintu M., Tolias C., Samuels M., Mazarakis N.K., Eravci M., Stewart N., Critchley G., Giamas G. (2022). Definition of an Inflammatory Biomarker Signature in Plasma-Derived Extracellular Vesicles of Glioblastoma Patients. Biomedicines.

[88] Nahand J.S., Vandchali N.R., Darabi H., Doroudian M., Banafshe H.R., Moghoofei M., Babaei F., Salmaninejad A., Mirzaei H. (2020). Exosomal MicroRNAs: Novel Players in Cervical Cancer. Epigenomics.

[89] Azhdari M.H., Goodarzi N., Doroudian M., MacLoughlin R. (2022). Molecular Insight into the Therapeutic Effects of Stem Cell-Derived Exosomes in Respiratory Diseases and the Potential for Pulmonary Delivery. Int. J. Mol. Sci..

[90] Mahmoudi K., Ezrin A., Hadjipanayis C. (2015). Small extracellular vesicles as tumor biomarkers for glioblastoma. Mol. Asp. Med..

[91] Mondal A., Kumari Singh D., Panda S., Shiras A. (2017). Extracellular Vesicles as Modulators of Tumor Microenvironment and Disease Progression in Glioma. Front. Oncol..

[92] Tkach M., Théry C. (2016). Communication by Extracellular Vesicles: Where We Are and Where We Need to Go. Cell.

[93] Yáñez-Mó M., Siljander P.R.-M., Andreu Z., Bedina Zavec A., Borràs F.E., Buzas E.I., Buzas K., Casal E., Cappello F., Carvalho J. (2015). Biological properties of extracellular vesicles and their physiological functions. J. Extracell. Vesicles.

[94] Hanjani N.A., Esmaelizad N., Zanganeh S., Gharavi A.T., Heidarizadeh P., Radfar M., Omidi F., MacLoughlin R., Doroudian M. (2022). Emerging role of exosomes as biomarkers in cancer treatment and diagnosis. Crit. Rev. Oncol. Hematol..

[95] Gharavi A.T., Hanjani N.A., Movahed E., Doroudian M. (2022). The role of macrophage subtypes and exosomes in immunomodulation. Cell Mol. Biol. Lett..

[96] Del Bene M., Osti D., Faletti S., Beznoussenko G.V., DiMeco F., Pelicci G. (2022). Extracellular vesicles: The key for precision medicine in glioblastoma. Neuro-Oncology.

[97] Doroudian M., Zanganeh S., Abbasgholinejad E., Donnelly S.C. (2023). Nanomedicine in Lung Cancer Immunotherapy. Front. Bioeng. Biotechnol..

[98] Tataranu L.G., Turliuc S., Kamel A., Rizea R.E., Dricu A., Staicu G.-A., Baloi S.C., Rodriguez S.M.B., Manole A.I.M. (2024). Glioblastoma Tumor Microenvironment: An Important Modulator for Tumoral Progression and Therapy Resistance. Curr. Issues Mol. Biol..

[99] Soung Y., Ford S., Zhang V., Chung J. (2017). Exosomes in Cancer Diagnostics. Cancers..

[100] Whitehead C.A., Kaye A.H., Drummond K.J., Widodo S.S., Mantamadiotis T., Vella L.J., Stylli S.S. (2020). Extracellular vesicles and their role in glioblastoma. Crit. Rev. Clin. Lab. Sci..

[101] Koch C.J., Lustig R.A., Yang X.-Y., Jenkins W.T., Wolf R.L., Martinez-Lage M., Desai A., Williams D., Evans S.M. (2014). Microvesicles as a Biomarker for Tumor Progression versus Treatment Effect in Radiation/Temozolomide-Treated Glioblastoma Patients. Transl. Oncol..

[102] Evans S.M., Putt M., Yang X.-Y., Lustig R.A., Martinez-Lage M., Williams D., Desai A., Wolf R., Brem S., Koch C.J. (2016). Initial evidence that blood-borne microvesicles are biomarkers for recurrence and survival in newly diagnosed glioblastoma patients. J. Neuro-Oncol..

[103] Osti D., Del Bene M., Rappa G., Santos M., Matafora V., Richichi C., Faletti S., Beznoussenko G.V., Mironov A., Bachi A. (2019). Clinical Significance of Extracellular Vesicles in Plasma from Glioblastoma Patients. Clin. Cancer Res..

[104] Galindo-Hernandez O., Villegas-Comonfort S., Candanedo F., González-Vázquez M.-C., Chavez-Ocaña S., Jimenez-Villanueva X., Sierra-Martinez M., Salazar E.P. (2013). Elevated Concentration of Microvesicles Isolated from Peripheral Blood in Breast Cancer Patients. Arch. Med. Res..

[105] Döring K., Malinova V., Bettag C., Rohde V., Schulz M., Menck K., Bleckmann A., Binder C., Büntzel J. (2024). The Diagnostic Potential of Extracellular Vesicles Derived from the Blood Plasma of Glioblastoma Patients. In Vivo.

[106] Rosas-Alonso R., Colmenarejo-Fernández J., Pernía O., Burdiel M., Rodríguez-Antolín C., Losantos-García I., Rubio T., Moreno-Velasco R., Esteban-Rodríguez I., Martínez-Marín V. (2024). Evaluation of the clinical use of MGMT methylation in extracellular vesicle-based liquid biopsy as a tool for glioblastoma patient management. Sci. Rep..

[107] Pan B.-T., Johnstone R.M. (1983). Fate of the transferrin receptor during maturation of sheep reticulocytes in vitro: Selective externalization of the receptor. Cell.

[108] Cheng J., Meng J., Zhu L., Peng Y. (2020). Exosomal noncoding RNAs in Glioma: Biological functions and potential clinical applications. Mol. Cancer.

[109] Heydari R., Koohi F., Rasouli M., Rezaei K., Abbasgholinejad E., Bekeschus S., Doroudian M. (2023). Exosomes as Rheumatoid Arthritis Diagnostic Biomarkers and Therapeutic Agents. Vaccines.

[110] Dai J., Su Y., Zhong S., Cong L., Liu B., Yang J., Tao Y., He Z., Chen C., Jiang Y. (2020). Exosomes: Key players in cancer and potential therapeutic strategy. Signal Transduct. Target. Ther..

[111] Luo H., Zhang H., Mao J., Cao H., Tao Y., Zhao G., Zhang Z., Zhang N., Liu Z., Zhang J. (2023). Exosome-based nanoimmunotherapy targeting TAMs, a promising strategy for glioma. Cell Death Dis..

[112] Chen J., Shan S., Xia B., Zhang L., Liang X. (2023). Brain-Targeted Exosomes-Based Drug Delivery System to Overcome the Treatment Bottleneck of Brainstem Glioma. Adv. Funct. Mater..

[113] Vaidya M., Sugaya K. (2020). DNA Associated with Circulating Exosomes as a Biomarker for Glioma. Genes.

[114] Mukherjee S., Pillai P.P. (2022). Current insights on extracellular vesicle-mediated glioblastoma progression: Implications in drug resistance and epithelial-mesenchymal transition. Biochim. Biophys. Acta-Gen. Subj..

[115] Cumba Garcia L.M., Bouchal S.M., Bauman M.M.J., Parney I.F. (2023). Advancements and Technical Considerations for Extracellular Vesicle Isolation and Biomarker Identification in Glioblastoma. Neurosurgery.

[116] Khatami S.H., Karami N., Taheri-Anganeh M., Taghvimi S., Tondro G., Khorsand M., Soltani Fard E., Sedighimehr N., Kazemi M., Rahimi Jaberi K. (2023). Exosomes: Promising Delivery Tools for Overcoming Blood-Brain Barrier and Glioblastoma Therapy. Mol. Neurobiol..

[117] Yang Q., Wei B., Peng C., Wang L., Li C. (2022). Identification of serum exosomal miR-98–5p, miR-183–5p, miR-323–3p and miR-19b-3p as potential biomarkers for glioblastoma patients and investigation of their mechanisms. Curr. Res. Transl. Med..

[118] Jiang Y., Wang F., Wang K., Zhong Y., Wei X., Wang Q., Zhang H. (2022). Engineered Exosomes: A Promising Drug Delivery Strategy for Brain Diseases. Curr. Med. Chem..

[119] Wang L., Wang D., Ye Z., Xu J. (2023). Engineering Extracellular Vesicles as Delivery Systems in Therapeutic Applications. Adv. Sci..

[120] Kar R., Dhar R., Mukherjee S., Nag S., Gorai S., Mukerjee N., Mukherjee D., Vatsa R., Chandrakanth Jadhav M., Ghosh A. (2023). Exosome-Based Smart Drug Delivery Tool for Cancer Theranostics. ACS Biomater. Sci. Eng..

[121] Bian X., Xiao Y.-T., Wu T., Yao M., Du L., Ren S., Wang J. (2019). Microvesicles and chemokines in tumor microenvironment: Mediators of intercellular communications in tumor progression. Mol. Cancer..

[122] Rackles E., Lopez P.H., Falcon-Perez J.M. (2022). Extracellular vesicles as source for the identification of minimally invasive molecular signatures in glioblastoma. Semin. Cancer Biol..

[123] Menck K., Sivaloganathan S., Bleckmann A., Binder C. (2020). Microvesicles in Cancer: Small Size, Large Potential. Int. J. Mol. Sci..

[124] Simionescu N., Nemecz M., Petrovici A.-R., Nechifor I.S., Buga R.-C., Dabija M.G., Eva L., Georgescu A. (2022). Microvesicles and Microvesicle-Associated microRNAs Reflect Glioblastoma Regression: Microvesicle-Associated miR-625-5p Has Biomarker Potential. Int. J. Mol. Sci..

[125] Baillie J.K., Barnett M.W., Upton K.R., Gerhardt D.J., Richmond T.A., De Sapio F., Brennan P.M., Rizzu P., Smith S., Fell M. (2011). Somatic retrotransposition alters the genetic landscape of the human brain. Nature..

[126] Ma S., Zhou M., Xu Y., Gu X., Zou M., Abudushalamu G., Yao Y., Fan X., Wu G. (2023). Clinical application and detection techniques of liquid biopsy in gastric cancer. Mol. Cancer.

[127] Zhou Y., Tao L., Qiu J., Xu J., Yang X., Zhang Y., Tian X., Guan X., Cen X., Zhao Y. (2024). Tumor biomarkers for diagnosis, prognosis and targeted therapy. Signal Transduct. Target. Ther..

[128] Zanganeh S., Abbasgholinejad E., Doroudian M., Esmaelizad N., Farjadian F., Benhabbour S.R. (2023). The Current Landscape of Glioblastoma Biomarkers in Body Fluids. Cancers.

[129] Zhang H., Yuan F., Qi Y., Liu B., Chen Q. (2021). Circulating Tumor Cells for Glioma. Front. Oncol..

[130] van Schaijik B., Wickremesekera A.C., Mantamadiotis T., Kaye A.H., Tan S.T., Stylli S.S., Itinteang T. (2019). Circulating tumor stem cells and glioblastoma: A review. J. Clin. Neurosci..

[131] Liu T., Xu H., Huang M., Ma W., Saxena D., Lustig R.A., Alonso-Basanta M., Zhang Z., O’Rourke D.M., Zhang L. (2018). Circulating Glioma Cells Exhibit Stem Cell-like Properties. Cancer Res..

[132] Lynch D., Powter B., Po J.W., Cooper A., Garrett C., Koh E.-S., Sheridan M., van Gelder J., Darwish B., Mckechnie S. (2020). Isolation of Circulating Tumor Cells from Glioblastoma Patients by Direct Immunomagnetic Targeting. Appl. Sci..

[133] MacArthur K.M., Kao G.D., Chandrasekaran S., Alonso-Basanta M., Chapman C., Lustig R.A., Wileyto E.P., Hahn S.M., Dorsey J.F. (2014). Detection of Brain Tumor Cells in the Peripheral Blood by a Telomerase Promoter-Based Assay. Cancer Res..

[134] Müller C., Holtschmidt J., Auer M., Heitzer E., Lamszus K., Schulte A., Matschke J., Langer-Freitag S., Gasch C., Stoupiec M. (2014). Hematogenous dissemination of glioblastoma multiforme. Sci. Transl. Med..

[135] Sullivan J.P., Nahed B.V., Madden M.W., Oliveira S.M., Springer S., Bhere D., Chi A.S., Wakimoto H., Rothenberg S.M., Sequist L.V. (2014). Brain Tumor Cells in Circulation Are Enriched for Mesenchymal Gene Expression. Cancer Discov..

[136] Cirkel G.A., Gadellaa-van Hooijdonk C.G., Koudijs M.J., Willems S.M., Voest E.E. (2014). Tumor Heterogeneity and Personalized Cancer Medicine: Are we Being Outnumbered?. Future Oncol..

[137] Ni X., Castanares M., Mukherjee A., Lupold S.E. (2011). Nucleic Acid Aptamers: Clinical Applications and Promising New Horizons. Curr. Med. Chem..

[138] Kichkailo A.S., Narodov A.A., Komarova M.A., Zamay T.N., Zamay G.S., Kolovskaya O.S., Erakhtin E.E., Glazyrin Y.E., Veprintsev D.V., Moryachkov R.V. (2023). Development of DNA aptamers for visualization of glial brain tumors and detection of circulating tumor cells. Mol. Ther. Nucleic. Acids.

[139] Rodriguez S.M.B., Kamel A., Ciubotaru G.V., Onose G., Sevastre A.-S., Sfredel V., Danoiu S., Dricu A., Tataranu L.G. (2023). An Overview of EGFR Mechanisms and Their Implications in Targeted Therapies for Glioblastoma. Int. J. Mol. Sci..

[140] Joosse S.A., Pantel K. (2015). Tumor-Educated Platelets as Liquid Biopsy in Cancer Patients. Cancer Cell.

[141] Gatto L., Franceschi E., Di Nunno V., Tosoni A., Lodi R., Brandes A.A. (2021). Liquid Biopsy in Glioblastoma Management: From Current Research to Future Perspectives. Oncologist.

[142] Stratz C., Nührenberg T.G., Binder H., Valina C.M., Trenk D., Hochholzer W., Neumann F., Fiebich B.L. (2012). Micro-array profiling exhibits remarkable intra-individual stability of human platelet, m.i.c.r.o.-R.N.A. Thromb. Haemost..

[143] Nilsson R.J.A., Balaj L., Hulleman E., van Rijn S., Pegtel D.M., Walraven M., Widmark A., Gerritsen W.R., Verheul H.M., Vandertop W.P. (2011). Blood platelets contain tumor-derived RNA biomarkers. Blood.

[144] Best M.G., Wesseling P., Wurdinger T. (2018). Tumor-Educated Platelets as a Noninvasive Biomarker Source for Cancer Detection and Progression Monitoring. Cancer Res..

[145] Sol N., in ‘t Veld S.G.J.G., Vancura A., Tjerkstra M., Leurs C., Rustenburg F., Schellen P., Verschueren H., Post E., Zwaan K. (2020). Tumor-Educated Platelet RNA for the Detection and (Pseudo)progression Monitoring of Glioblastoma. Cell Rep. Med..

[146] Zhou Y., Yang L., Zhang X., Chen R., Chen X., Tang W., Zhang M. (2019). Identification of Potential Biomarkers in Glioblastoma through Bioinformatic Analysis and Evaluating Their Prognostic Value. BioMed Res. Int..

[147] Seyhan A.A. (2024). Circulating Liquid Biopsy Biomarkers in Glioblastoma: Advances and Challenges. Int. J. Mol. Sci..

